# Ferric uptake regulator (Fur) binds a [2Fe-2S] cluster to regulate intracellular iron homeostasis in *Escherichia coli*

**DOI:** 10.1016/j.jbc.2023.104748

**Published:** 2023-04-24

**Authors:** Chelsey R. Fontenot, Huangen Ding

**Affiliations:** Department of Biological Sciences, Louisiana State University, Baton Rouge, Louisiana, USA

**Keywords:** gene expression regulation, Ferric uptake regulator, Fur-box, iron–sulfur cluster, iron homeostasis

## Abstract

Intracellular iron homeostasis in bacteria is primarily regulated by ferric uptake regulator (Fur). It has been postulated that when intracellular free iron content is elevated, Fur binds ferrous iron to downregulate the genes for iron uptake. However, the iron-bound Fur had not been identified in any bacteria until we recently found that *Escherichia coli* Fur binds a [2Fe-2S] cluster, but not a mononuclear iron, in *E. coli* mutant cells that hyperaccumulate intracellular free iron. Here, we report that *E. coli* Fur also binds a [2Fe-2S] cluster in wildtype *E. coli* cells grown in M9 medium supplemented with increasing concentrations of iron under aerobic growth conditions. Additionally, we find that binding of the [2Fe-2S] cluster in Fur turns on its binding activity for specific DNA sequences known as the Fur-box and that removal of the [2Fe-2S] cluster from Fur eliminates its Fur-box binding activity. Mutation of the conserved cysteine residues Cys-93 and Cys-96 to Ala in Fur results in the Fur mutants that fail to bind the [2Fe-2S] cluster, have a diminished binding activity for the Fur-box *in vitro*, and are inactive to complement the function of Fur *in vivo*. Our results suggest that Fur binds a [2Fe-2S] cluster to regulate intracellular iron homeostasis in response to elevation of intracellular free iron content in *E. coli* cells.

Ferric uptake regulator (Fur) is a global transcription factor that regulates intracellular iron homeostasis, oxidative stress response, and virulence in bacteria (1, 2). Since the discovery of Fur in *Escherichia coli* (3), it has been hypothesized that when intracellular free iron content is elevated, Fur binds ferrous iron to form an iron-bound Fur which in turn binds specific DNA sequences known as the Fur-box to downregulate the genes for iron uptake and indirectly upregulate the genes for iron storage (4, 5, 6, 7, 8, 9). Structural studies have shown that *E. coli* Fur (10) and its homologs from other bacteria (11, 12, 13, 14, 15, 16) exist as a homodimer or tetramer with each monomer containing an N-terminal DNA binding domain and C-terminal dimerization domain. Each Fur monomer has three putative metal binding sites (10, 13). Site 1 (coordinated by His-87, Asp-89, Glu-108, and His-125, residue numbers in *E. coli* Fur) is localized within the dimerization domain. Site 2 (coordinated by His-33, Glu-81, His-88, and His-90) connects the DNA binding domain and the dimerization domain. Site 3 (coordinated by Cys-93, Cys-96, and Cys-133) is at the C-terminal end of the dimerization domain. In purified Fur proteins, site 1 and site 2 are often occupied by Zn(II) (11, 13, 17). However, while Fur can be reconstituted with Fe(II) or other divalent cations *in vitro* (18, 19, 20, 21), the iron-bound Fur had not been identified in any bacteria until our recent study showing that *E. coli* Fur binds a [2Fe-2S] cluster (22).

In searching for the proposed iron-bound Fur *in vivo*, we expressed recombinant *E. coli* Fur in the *E. coli* mutant cells in which intracellular free iron content was elevated due to deletion of the iron–sulfur cluster assembly proteins IscA and SufA (23) and found that purified *E. coli* Fur had a bright red color. The UV-Vis, electron paramagnetic resonance, and Mössbauer spectroscopy studies showed that purified red Fur contains a [2Fe-2S] cluster, but not a mononuclear iron (22). Quantification of iron and sulfur contents combining with the UV-visible absorption spectrum measurements revealed that about 32% of red Fur protein purified from the *E. coli iscA/sufA* mutant cells grown in LB medium under aerobic conditions binds a [2Fe-2S] cluster. In contrast, only about 4% of *E. coli* Fur purified from wildtype *E. coli* cells binds a [2Fe-2S] cluster (22), suggesting that the occupancy of the [2Fe-2S] cluster in Fur increases in response to elevation of intracellular free iron content. Site-directed mutagenesis studies further revealed that *E. coli* Fur binds the [2Fe-2S] cluster *via* the conserved Cys-93, Cys-96, and Cys-133 as mutation of Cys-93, Cys-96, or Cys-133 to Ala results in the Fur mutants that fail to bind the [2Fe-2S] cluster in *E. coli* cells (22).

To investigate whether Fur can also bind a [2Fe-2S] cluster in wildtype *E. coli* cells in response to elevation of intracellular free iron content, here we have expressed Fur in two commonly used wildtype *E. coli* strains (MC4100 and GC4468) grown in M9 medium supplemented with increasing concentrations of iron under aerobic growth conditions and purified the Fur proteins from the cells. The results showed that *E. coli* Fur proportionally binds a [2Fe-2S] cluster in wildtype *E. coli* cells as the iron concentration in M9 medium gradually increases (up to 1.0 μM iron in M9 medium). Importantly, the *in vitro* DNA binding assays revealed that binding of the [2Fe-2S] cluster in *E. coli* Fur turns on its Fur-box binding activity and that removal of the [2Fe-2S] cluster from Fur eliminates the Fur-box binding activity. Furthermore, mutation of Cys-93 or Cys-96 to Ala results in the Fur mutants that do not bind the [2Fe-2S] cluster, have a diminished binding activity for the Fur-box *in vitro*, and are inactive to complement the function of Fur *in vivo*. The results led us to propose that *E. coli* Fur reversibly binds a [2Fe-2S] cluster to regulate intracellular-iron homeostasis in response to elevation of intracellular free iron content in *E. coli*.

## Results

### *E. coli* Fur binds a [2Fe-2S] cluster in wildtype *E. coli* cells grown in M9 medium supplemented with iron

**Figure 1:**
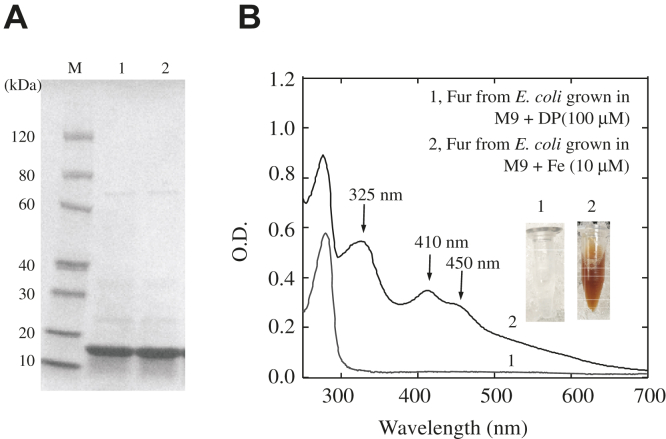
***E. coli* Fur binds a [2Fe-2S] cluster in wildtype *E. coli* cells grown in M9 medium supplemented with iron.***A*, SDS-PAGE gel of Fur proteins purified from *E. coli* cells grown in M9 medium supplemented with 2,2′-dipyridyl (100 μM) (lane 1) or Fe(NH_4_)_2_(SO_4_)_2_ (10.0 μM) (lane 2). Lane M, PAGE-MASTER protein markers (GenScript co) with molecular weights. *B*, UV-Vis absorption spectra of Fur proteins purified from wildtype *E. coli* cells grown in M9 medium supplemented with either 2,2′-dipyridyl (100 μM) (spectrum 1) (Apo-Fur) or Fe(NH_4_)_2_(SO_4_)_2_ (10.0 μM) (Red-Fur) (spectrum 2) under aerobic growth conditions. Purified Fur proteins (50 μM) were in buffer containing NaCl (500 mM) and Tris (20 mM, pH 8.0). Insert is the photograph of purified Apo-Fur (1) and Red-Fur (2). Fur, ferric uptake regulator.

M9 medium is known to be iron deficient (∼0.05 μM total iron) (24). When *E. coli* cells are grown in M9 medium supplemented with 10 μM iron, the intracellular free iron content is elevated, and Fur becomes an active repressor to downregulate the genes for iron uptake (25). To test whether *E. coli* Fur can bind a [2Fe-2S] cluster in wildtype *E. coli* cells with an elevated intracellular free iron content, we expressed *E. coli* Fur in an *E. coli* wildtype (MC4100) cells grown in M9 medium supplemented with either 2,2′-dipyridyl (100 μM) (to deplete intracellular free iron content) or Fe(NH_4_)_2_(SO_4_)_2_ (10 μM) (to replete intracellular free iron content) (25). Fur was then purified from the *E. coli* cells (Fig. 1*A*), as described previously (22).

Figure 1*B* shows that *E. coli* Fur purified from wildtype *E. coli* cells grown in M9 medium supplemented with 2,2′-dipyridyl (100 μM) was colorless and had no absorption peaks in the visible range (spectrum 1) (**Apo-Fur**). In contrast, Fur purified from wildtype *E. coli* cells grown in M9 medium supplemented with Fe(NH_4_)_2_(SO_4_)_2_ (10 μM) had a bright red color (**Red-Fur**) and distinct absorption peaks at 325 nm, 410 nm, and 450 nm (spectrum 2), indicative of the [2Fe-2S] cluster binding in Fur (22, 26). The [2Fe-2S] cluster in Red-Fur was confirmed by the electron paramagnetic resonance and Mössbauer spectroscopy as described previously (22). Using the extinction coefficient of 10 mM^−1^ cm^−1^ at 410 nm for the [2Fe-2S] cluster in *E. coli* Fur (22), we estimated that the occupancy of the [2Fe-2S] cluster in purified Red-Fur was 36 ± 5% (n = 3). The iron and sulfur content analyses showed that Red-Fur contained 0.66 ± 0.15 iron and 0.43 ± 0.21 sulfide atoms per Fur monomer, while Apo-Fur had no detectable amounts of iron and sulfide, consistent with the estimated occupancy of the [2Fe-2S] cluster in these proteins. We also analyzed the Zn(II) contents of the purified Fur proteins and found that Apo-Fur and Red-Fur contained 0.47 ± 0.12 and 0.51 ± 0.15 Zn(II) atoms per Fur monomer (n = 3), respectively, indicating that both Apo-Fur and Red-Fur had tightly bound Zn(II) (11, 13, 17). Thus, *E. coli* Fur binds a [2Fe-2S] cluster in response to elevation of intracellular free iron content in wildtype *E. coli* cells.

### Binding of a [2Fe-2S] cluster in Fur turns on its Fur-box binding activity

**Figure 2:**
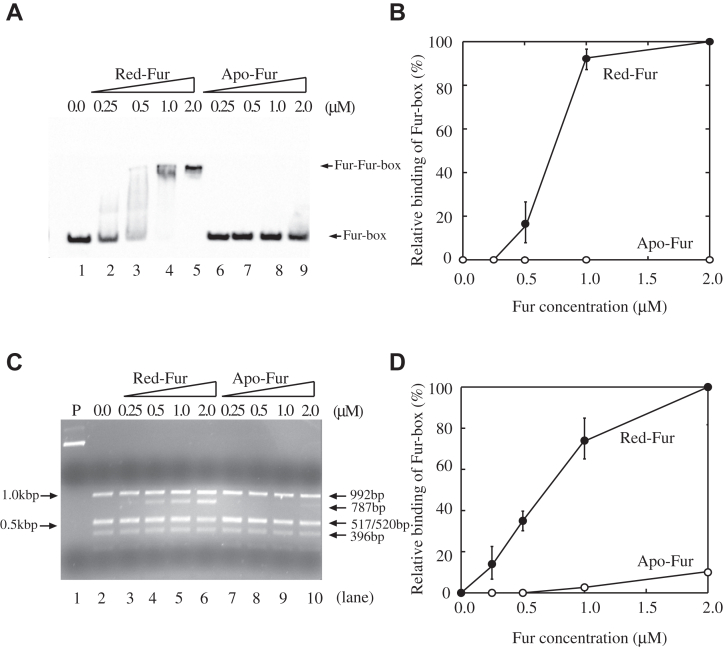
**The Fur-box binding activity of purified Red-Fur and Apo-Fur.***A*, band shift assays of Red-Fur and Apo-Fur. Biotin-labeled Fur-box DNA (0.7 nM) was incubated with the indicated concentrations of Red-Fur or Apo-Fur. Lane 1, no Fur protein. Lanes 2 to 5, biotin-labeled Fur-box DNA (0.7 nM) was incubated with 0.25 μM, 0.5 μM, 1.0 μM, and 2.0 μM Red-Fur, respectively. Lanes 6 to 9, biotin-labeled Fur-box DNA (0.7 nM) was incubated with 0.25 μM, 0.5 μM, 1.0 μM, and 2.0 μM Apo-Fur, respectively. *B*, relative binding activity of Red-Fur and Apo-Fur based on the band shift assays. The intensities of the Fur/Fur-box bands in shown (*A*) were quantified using ImageJ and plotted as a function of the Fur concentrations. Data represent the averages ± standard deviations from three independent experiments. *C*, the restriction site protection assays of Red-Fur and Apo-Fur. pUC18-*iuc* (3.2 nM) was preincubated with increasing concentrations of Red-Fur and Apo-Fur, followed by digestion with *Hin*fI (1 unit) at 37 °C for 10 min. The digested DNA products were separated by 1.5% agarose gel electrophoresis. Lane 1, pUC18-*iuc* only. Lane 2, no Fur protein was added. Lanes 3 to 6, pUC18-*iuc* (3.2 nM) was preincubated with 0.25 μM, 0.5 μM, 1.0 μM, and 2.0 μM Red-Fur, respectively. Lanes 7 to 10, pUC18-*iuc* was preincubated with 0.25 μM, 0.5 μM, 1.0 μM, and 2.0 μM Apo-Fur, respectively. *D*, relative binding activity of Red-Fur and Apo-Fur based on the restriction site protection assays. The intensities of the DNA band at 787 bp shown in (*C*) were quantified using ImageJ and plotted as a function of the Fur concentrations. Data represent the averages ± standard deviations from three independent experiments.

*E. coli* Fur controls the expression of its target genes by binding to specific DNA sequences known as the Fur-box (4, 5, 6). To explore the Fur-box binding activity of purified Apo-Fur and Red-Fur, a biotin-labeled DNA fragment (110 bps) containing a consensus Fur-box (5′-TATAATGATACGCATTATC-3′) (4) was prepared and incubated with increasing concentrations of Apo-Fur or Red-Fur, followed by the electrophoretic mobility shift assays. Figure 2*A* shows that while Apo-Fur (up to 2.0 μM) had very little or no binding of the Fur-box, 1.0 μM Red-Fur was sufficient to completely shift the Fur-box (0.7 nM). The Fur/Fur-box complex bands in Figure 2*A* were quantified using ImageJ (NIH) and plotted as a function of the Fur concentrations in the incubation solutions. Unlike Apo-Fur, Red-Fur had a strong binding activity for the Fur-box (Fig. 2*B*). Since only about 36% of Red-Fur contained a [2Fe-2S] cluster (Fig. 1*B*), the [2Fe-2S] cluster-bound Fur likely has a much stronger binding activity for the Fur-box.

To further explore the Fur-box binding activity of Apo-Fur and Red-Fur, we used the restriction site protection assay. The promoter region of the operon *iucABCD* which encodes the enzymes for biosynthesis of siderophore aerobactin (27) has a consensus Fur-box sequence (5′-GAGAATCATTAGCATTCGC-3′) (4) which contains the restriction *hin*fI site (5′-GANTC-3′) (18). Binding of Fur to the Fur-box protects the *hin*fI site from being cleaved by *Hin*fI (18). The restriction site protection assay has been applied to investigate the Fur-box binding activity of *E. coli* Fur after nitric oxide exposure (28), of the Co(II)-bound *Helicobacter pylori* Fur (21), and of the Mn(II)-bound *Aliivibrio salmonicida* Fur (29). For the experiments, the promoter sequence of the operon *iucABCD* was synthesized (GenScript co) and inserted into plasmid pUC19 to create pUC19-*iuc* (Fig. S1*A*). After pUC19-*iuc* was digested with restriction enzyme *Hin*fI, four major DNA fragments (992 bp, 517 bp/520 bp, 396 bp, and 267 bp) were produced. In the presence of Red-Fur, the *hin*fI site in the Fur-box was protected from the *Hin*fI digestion and a new DNA fragment at 787 bp appeared (Fig. S1*B*).

The pUC19-*iuc* plasmid was then preincubated with increasing concentrations of Apo-Fur or Red-Fur, followed by the *Hin*fI digestion. Figure 2*C* shows that 1.0 μM Red-Fur was sufficient to protect the Fur-box in pUC19-*iuc* from the *Hin*fI digestion (lane 5). In contrast, Apo-Fur (up to 2.0 μM) had very little or no protection for the Fur-box in pUC19-*iuc* (lane 10). The band intensities of the 787-bp DNA fragment were quantified using ImageJ (NIH) and plotted as a function of the Fur concentrations in the incubation solutions (Fig. 2*D*). Taken together, the results in Figure 2 demonstrate that unlike Apo-Fur, Red-Fur is active to bind the Fur-box *in vitro*.

### Red-Fur loses the Fur-box binding activity when the [2Fe-2S] cluster is removed

**Figure 3:**
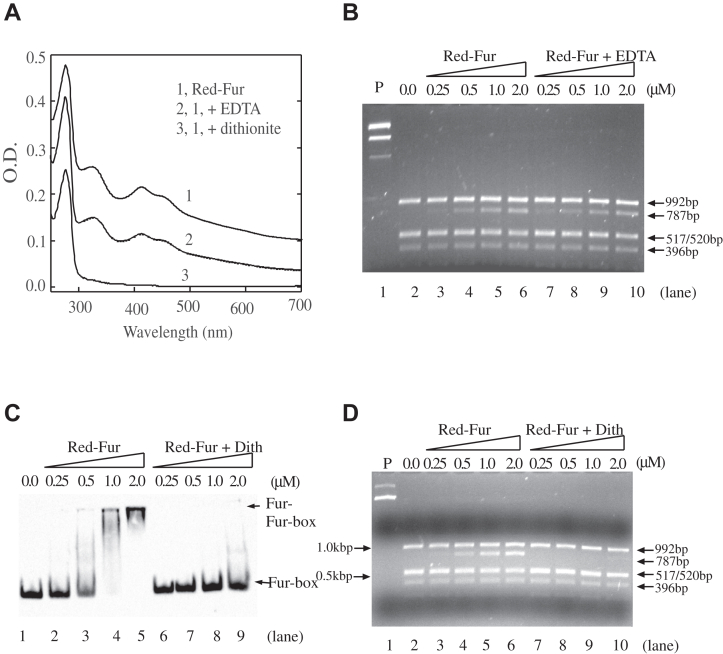
**Red-Fur loses its Fur-box binding activity upon removal of the [2Fe-2S] cluster.***A*, UV-Vis absorption spectra of Red-Fur after being treated with EDTA (0.1 mM) or sodium dithionite (4 mM), followed by the dialysis against buffer containing NaCl (500 mM) and Tris (20 mM, pH 8.0). Spectrum 1, purified Red-Fur (50 μM); spectrum 2, Red-Fur (50 μM) after being treated with EDTA and dialysis; spectrum 3, Red-Fur (50 μM) after being treated with sodium dithionite and dialysis. *B*, the restriction site protection assays of the EDTA-treated Red-Fur. Lane 1, pUC18-*iuc* (3.2 nM) only. Lane 2, no Fur protein was added before the *Hin*fI digestion. Lanes 3 to 6, pUC18-*iuc* (3.2 nM) was preincubated with 0.25 μM, 0.5 μM, 1.0 μM, and 2.0 μM Red-Fur, respectively, followed by the *Hin*fI digestion. Lanes 7 to 10, pUC18-*iuc* (3.2 nM) was preincubated with 0.25 μM, 0.5 μM, 1.0 μM, and 2.0 μM the EDTA-treated Red-Fur, respectively, followed by the *Hin*fI digestion. *C*, the Fur-box binding activity of Red-Fur after removal of the [2Fe-2S] cluster. Biotin-labeled Fur-box DNA (0.7 nM) was incubated with increasing concentrations of Red-Fur before and after removal of the [2Fe-2S] cluster. Lane 1, no Fur protein was added. Lanes 2 to 5, the biotin-labeled Fur-box DNA (0.7 nM) was incubated with 0.25 μM, 0.5 μM, 1.0 μM, and 2.0 μM Red-Fur, respectively. Lanes 6 to 9, the biotin-labeled Fur-box DNA (0.7 nM) was incubated with 0.25 μM, 0.5 μM, 1.0 μM, and 2.0 μM Fur without the [2Fe-2S] cluster, respectively. *D*, the restriction site protection assays of Red-Fur after removal of the [2Fe-2S] cluster. pUC18-*iuc* (3.2 nM) was preincubated with increasing concentrations of Red-Fur with or without the [2Fe-2S] cluster, followed by digestion with *Hin*fI at 37 ^°^C for 10 min. The digested DNA products were separated on 1.5% agarose gel electrophoresis. Lane 1, pUC18-*iuc* (3.2 nM) only. Lane 2, no Fur protein was added. Lanes 3 to 6, pUC18-*iuc* (3.2 nM) was preincubated with 0.25 μM, 0.5 μM, 1.0 μM, and 2.0 μM Red-Fur, respectively. Lanes 7 to 10, pUC18-*iuc* (3.2 nM) was preincubated with 0.25 μM, 0.5 μM, 1.0 μM, and 2.0 μM Fur without the [2Fe-2S] cluster, respectively. The data are representative of three independent experiments. Fur, Ferric uptake regulator.

Previous studies indicated that *E. coli* Fur became active to bind the Fur-box upon binding of Fe(II) or other divalent cations *in vitro* (18, 20). The dissociation constant of the mononuclear iron binding in *E. coli* Fur is in the range from 1.2 μM to 55 μM (19, 20, 21). Therefore, it is plausible that some mononuclear iron might bind to Red-Fur and contribute to the Fur-box binding activity. To remove any potential mononuclear iron from Red-Fur, the protein was treated with an iron chelator EDTA (0.1 mM), followed by the dialysis against buffer containing NaCl (500 mM) and Tris (20 mM, pH 8.0) at 4 ^°^C. EDTA (0.1 mM) was previously used to remove mononuclear iron from *E. coli* Fur (18). When Red-Fur was treated with EDTA (0.1 mM), the UV-Vis absorption spectrum of Red-Fur was not changed (Fig. 3*A*, spectrum 2), indicating that the [2Fe-2S] cluster in Fur was stable in the presence of EDTA (0.1 mM). This result was further confirmed by the iron and sulfide content analyses of Red-Fur before and after the EDTA treatment. The EDTA-treated Red-Fur was then subjected to the restriction site protection assay. Figure 3*B* shows that the EDTA treatment did not affect the Fur-box binding activity of Red-Fur, suggesting that mononuclear iron binding, if any, had no contributions to the Fur-box binding activity of Red-Fur.

Next, we sought to remove the [2Fe-2S] cluster from Red-Fur. Previously, we found that while the oxidized [2Fe-2S] cluster in *E. coli* Fur is stable, the reduced [2Fe-2S] cluster in *E. coli* Fur quickly releases ferrous iron and sulfide to form apo-form Fur (26). Therefore, we reduced Red-Fur with freshly prepared sodium dithionite in Tris (20 mM, pH 8.0), followed by the dialysis against buffer containing NaCl (500 mM) and Tris (20 mM, pH 8.0) at 4 ^°^C. Figure 3*A* shows that Red-Fur lost the [2Fe-2S] cluster upon reduction of the cluster and dialysis (spectrum 3), as reported previously (26). Red-Fur before or after removal of the [2Fe-2S] cluster was then subjected to the band shift assay (Fig. 3*C*) and the restriction site protection assay (Fig. 3*D*). Evidently, removal of the [2Fe-2S] cluster from Red-Fur results in apo-Fur that fails to bind the Fur-box.

### *E. coli* Fur proportionally binds a [2Fe-2S] cluster in wildtype *E. coli* cells grown in M9 medium supplemented with increasing concentrations of iron

**Figure 4:**
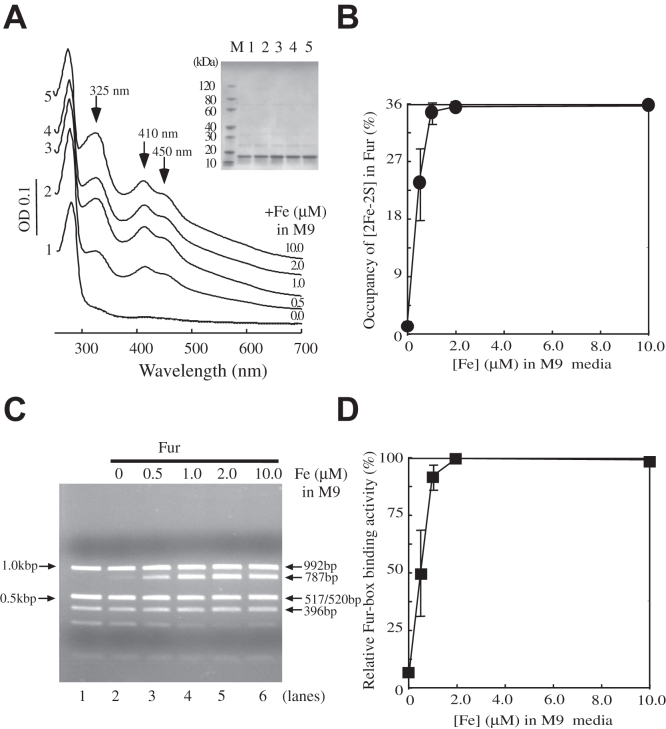
**Fur progressively binds a [2Fe-2S] cluster in wildtype *E. coli* cells grown in M9 medium supplemented with increasing concentrations of iron.***A*, UV-Vis absorption spectra of Fur proteins purified from wildtype *E. coli* cells grown in M9 medium supplemented with 0.0 (spectrum 1), 0.5 μM (spectrum 2), 1.0 μM (spectrum 3), 2.0 μM (spectrum 4), or 10.0 μM (spectrum 5) Fe(NH_4_)_2_(SO_4_)_2_ under aerobic growth conditions. Purified Fur proteins (50 μM) were in buffer containing NaCl (500 mM) and Tris (20 mM, pH 8.0). Insert is a photograph of the SDS-PAGE gel of purified Fur proteins. *B*, *E. coli* Fur binds the [2Fe-2S] cluster in wildtype *E. coli* cells in response to increasing concentrations of iron in M9 medium. The [2Fe-2S] cluster occupancies of Fur proteins purified from wildtype *E. coli* cells grown in M9 medium supplemented with 0.0 μM, 0.5 μM, 1.0 μM, 2.0 μM, and 10.0 μM Fe(NH_4_)_2_(SO_4_)_2_, respectively, were calculated and plotted as a function of the iron concentrations in M9 medium. Data represent the averages ± standard deviations from three independent experiments. *C*, the restriction site protection assays of Fur proteins. pUC19-*iuc* (3.2 nM) was preincubated with Fur proteins (1.0 μM) purified from wildtype *E. coli* cells grown in M9 medium supplemented with indicated concentrations of iron, followed by digestion with *Hin*fI at 37 ^°^C for 10 min. Lane 1, pUC19-*iuc* (3.2 nM) was digested with *Hin*fI without any Fur proteins. Lanes 2 to 6, pUC19-*iuc* (3.2 nM) was preincubated with Fur proteins (1.0 μM) purified from wildtype *E. coli* cells grown in M9 medium supplemented with 0.0 μM, 0.5 μM, 1.0 μM, 2.0 μM, and 10.0 μM Fe(NH_4_)_2_(SO_4_)_2_, respectively, followed by the *Hin*fI digestion. *D*, the relative Fur-box binding activities of Fur proteins purified from wildtype *E. coli* cells grown in M9 medium supplemented with increasing concentrations of iron. The intensities of the DNA band at 787 bp shown in (*C*) were plotted as a function of the iron concentrations in M9 medium. Data represent the averages ± standard deviations from three independent experiments. Fur, ferric uptake regulator.

Attempts to reconstitute the [2Fe-2S] cluster in apo-Fur *in vitro* were not successful, likely because *E. coli* Fur has a relatively weak binding activity for the [2Fe-2S] cluster (22). Here, we decided to explore the binding of the [2Fe-2S] cluster in Fur in wildtype *E. coli* cells grown in M9 medium supplemented with increasing concentrations of iron under aerobic growth conditions. Figure 4*A* shows that *E. coli* Fur purified from wildtype *E. coli* cells (MC4100) grown in M9 medium supplemented with no iron had only very small amplitudes of the absorption peaks at 325 nm, 410 nm, and 450 nm (spectrum 1). When M9 medium was supplemented with 0.5 μM iron, purified Fur had clear absorption peaks at 325 nm, 410 nm, and 450 nm (spectrum 2) with an estimated occupancy of the [2Fe-2S] cluster of about 23%. When M9 medium was supplemented with 1.0 μM iron, the occupancy of the [2Fe-2S] cluster in purified Fur reached about 36% (spectrum 3). Interestingly, further increase of the iron concentration in M9 medium did not increase the occupancy of the [2Fe-2S] cluster in Fur (Fig. 4*B*). A possible explanation could be that intracellular free iron content in wildtype *E. coli* cells is regulated by the active Fur in such that the maximum occupancy of the [2Fe-2S] cluster in Fur is limited to about 36% in the cells.

To examine whether the [2Fe-2S] cluster binding in Fur is limited to *E. coli* MC4100 which is known to have a number of mutations (30), we also expressed Fur in the *E. coli* GC4468 cells (Coli Genetic Stock Center, Yale University), another commonly used wildtype strain. When GC4468 cells expressing *E. coli* Fur were grown in M9 medium supplemented with increasing concentrations of iron (0–10 μM) under aerobic growth conditions, Fur also bound a [2Fe-2S] cluster with a maximum occupancy of the [2Fe-2S] cluster of about 36% (Fig. S2). Thus, *E. coli* Fur binds a [2Fe-2S] cluster in response to elevation of intracellular free iron content in wildtype *E. coli* cells under aerobic growth conditions.

The Fur proteins purified from wildtype *E. coli* cells (MC4100) grown in M9 medium supplemented with increasing concentrations of iron (0.0, 0.5, 1.0, 2.0, and 10.0 μM) were further subjected to the restriction site protection assay. Figure 4*C* shows that as the iron concentration in M9 medium was gradually increased, the Fur-box binding activity of the purified Fur was progressively increased and apparently saturated when the iron concentration in M9 medium was about 1.0 μM. The intensities of the 787-bp DNA fragment shown in Figure 4*C* were quantified and plotted as a function of the iron concentrations in M9 medium (Fig. 4*D*). The positive correlation between the [2Fe-2S] cluster occupancy (Fig. 4*B*) and the Fur-box binding activity (Fig. 4*D*) of purified Fur strongly suggests that *E. coli* Fur binds a [2Fe-2S] cluster and becomes active to bind the Fur-box in response to elevation of intracellular free iron content in *E. coli* cells.

### The Fur mutants that fail to bind a [2Fe-2S] cluster have a diminished binding activity for the Fur-box *in vitro* and are inactive to complement the function of Fur *in vivo*

**Figure 5:**
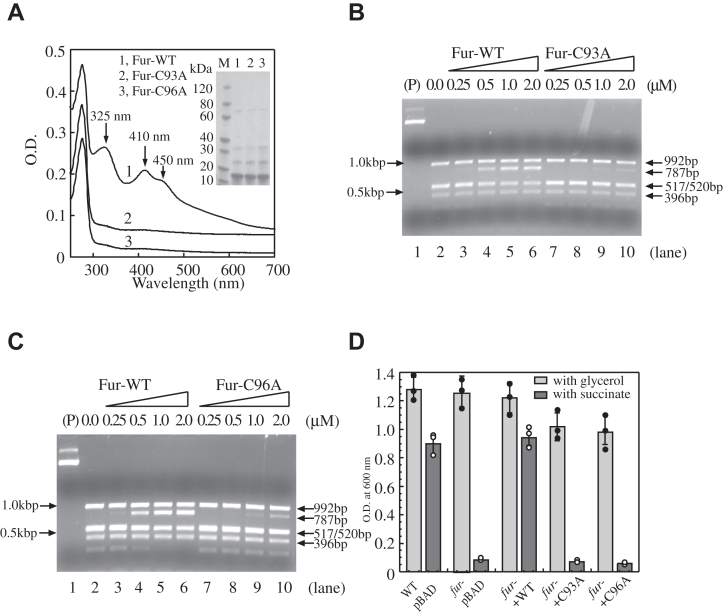
**Fur mutants that fail to bind the [2Fe-2S] cluster have a diminished Fur-box binding activity *in vitro* and are inactive *in vivo*.***A*, UV-Vis absorption spectra of wildtype *E. coli* Fur (spectrum 1) and Fur mutant Fur-C93A (spectrum 2) and Fur mutant Fur-C96A (spectrum 3). Each Fur protein was purified from wildtype *E. coli* cells grown in M9 medium supplemented with 2.0 μM Fe(NH_4_)_2_(SO_4_)_2_ under aerobic growth conditions. Purified Fur proteins (50 μM) were in buffer containing NaCl (500 mM) and Tris (20 mM, pH 8.0). Insert was a photograph of the SDS-PAGE. Lane M, molecular weight markers (M) (GenScript co). Lane 1, wildtype Fur. Lane 2, Fur-C93A. Lane 3, Fur-C96A. *B*, the Fur-box binding activity of Fur-C93A. Plasmid pUC19-*iuc* (3.2 nM) was preincubated with increasing concentrations of wildtype Fur or Fur-C93A, followed by the *Hin*fI digestion. Lane 1, pUC19-*iuc* (3.2 nM) only. Lane 2, pUC19-*iuc* was digested with *Hin*fI without any Fur proteins. Lanes 3 to 6, pUC19-*icu* (3.2 nM) was preincubated with 0.25 μM, 0.5 μM, 1.0 μM, and 2.0 μM Red-Fur, respectively, followed by the *Hin*fI digestion. Lanes 7 to 10, pUC19-*icu* (3.2 nM) was preincubated with 0.25 μM, 0.5 μM, 1.0 μM, and 2.0 μM Fur-C93A, respectively, followed by the *Hin*fI digestion. *C*, the Fur-box binding activity of Fur-C96A. Same as in (*B*), except Fur-C93A was replaced with Fur-C96A. *D*, the *in vivo* activity of *E. coli* Fur-C93A and Fur-C96A. pBAD expressing either wildtype *E. coli* Fur or Fur mutants Fur-C93A or Fur-C96A was introduced into the *E. coli fur* mutant cells. Overnight cell cultures were inoculated in M9 medium with either glycerol (0.4%) (gray bars) or succinate (0.4%) (dark bars). The cell growth was measured at absorbance of 600 nm after 20 h growth with aeration at 37 ^°^C. The data from three independent experiments are presented together with averages ± standard deviations. Fur, ferric uptake regulator.

To further explore the role of the [2Fe-2S] cluster in *E. coli* Fur for its Fur-box binding activity, we prepared Fur mutants C93A and C96A in which Cys-93 or Cys-96 was mutated to Ala from wildtype *E. coli* cells grown in M9 medium supplemented with 2.0 μM iron under aerobic growth conditions. Unlike wildtype Fur (Fig. 5*A*, spectrum 1), Fur-C93A and Fur-C96A did not bind any [2Fe-2S] clusters in *E. coli* cells (Fig. 5*A*, spectra 2 and 3) as reported previously (22). The iron and sulfide content analyses showed that both Fur mutants contained undetectable amounts of iron and sulfide, consistent with the notion that the Fur mutants fail to bind any [2Fe-2S] clusters (22). On the other hand, the Fur mutants C93A and C96A still contained 0.43 ± 0.10 and 0.46 ± 0.17 Zn(II) atoms per Fur monomer (n = 3), respectively, suggesting that mutations C93A and C96A did not significantly affect the Zn(II) binding in Fur. The Fur mutants Fur-C93A and Fur-C96A were then subjected to the restriction site protection assay. Figure 5, *B* and *C* show that both Fur-C93A and Fur-C96A had a diminished binding activity for the Fur-box. Similar results were also obtained from the band shift assay experiments (data not shown). We noticed that Fur-C93A and Fur-C96A appeared to have a weak binding activity for the Fur-box, possibly because that Fur-C93A and Fur-C96A have a protein conformation that is subtly different from Apo-Fur. Regardless, the results provide additional evidence for the notion that binding of a [2Fe-2S] cluster in Fur is crucial for its Fur-box binding activity.

To evaluate the *in vivo* activity of the Fur mutants Fur-C93A and Fur-C96A, we constructed an *E. coli* mutant strain in which gene *fur* was deleted using the one-step gene deletion procedure (31). While deletion of Fur has only a mild effect on cell growth in M9 medium using glycerol as carbon source, deletion of Fur results in a null-growth phenotype in M9 medium using succinate as only carbon source (32) (Fig. 5*D*). This is because deletion of gene *fur* results in an elevated expression of a small regulatory RNA RyhB which in turn downregulates expression of a group of iron-using proteins including succinate dehydrogenase in *E. coli* cells (33). Deficiency of succinate dehydrogenase leads to a null-growth phenotype of the *fur* mutant in M9 medium using succinate as only carbon source (32).

When plasmid expressing wildtype *E. coli* Fur was introduced into the *E. coli fur* mutant cells, the cell growth in M9 medium with succinate as carbon source was largely restored (Fig. 5*D*). However, plasmid expressing either Fur-C93A or Fur-C96A failed to restore the cell growth of the *E. coli fur* mutant in M9 medium with succinate as carbon source (Fig. 5*D*), suggesting that both Fur-C93A and Fur-C96A are inactive to complement the function of Fur *in vivo*.

## Discussion

Here we report that *E. coli* Fur binds a [2Fe-2S] cluster in wildtype *E. coli* cells grown in M9 medium supplemented with increasing concentrations of iron under aerobic growth conditions. The *in vitro* DNA binding activity assays show that binding of the [2Fe-2S] cluster in Fur turns on its Fur-box binding activity and that removal of the [2Fe-2S] cluster effectively turns off the Fur-box binding activity. Furthermore, the Fur mutants with mutation of Cys-93 or Cys-96 to Ala that fail to bind a [2Fe-2S] cluster (22) have a diminished binding activity for the Fur-box *in vitro* and are inactive to complement the function of Fur *in vivo*. The results led us to propose that *E. coli* Fur binds a [2Fe-2S] cluster to downregulate the genes for iron uptake in response to elevation of intracellular free iron content in *E. coli* cells.

Structural studies of Fur proteins from various bacteria (10, 11, 12, 13, 14, 15, 16) have revealed that Fur exists as a dimer or tetramer with each Fur monomer having three putative metal binding sites (10, 13). While some studies suggested that site 2 is structural and site 1 is regulatory (11), others argued that site 2 is regulatory and site 1 is auxiliary (13, 34). Regardless, it appears that Zn(II) binding at least at one of these sites (site 1 or site 2) is crucial for the stable structure and regulatory function of Fur (13, 17). It has also been reported that purified Fur can be reconstituted with excess Fe(II), Zn(II), Cu(II), Co(II), or Mn(II) *in vitro* with dissociation constants from 1.2 μM to 55 μM (18, 19, 20, 21). Considering the relatively weak binding affinities of Fur for these divalent cations, it was further postulated that many of these intracellular divalent cations may not be able to activate Fur *in vivo* because of their low intracellular concentrations (20). This may also explain why the iron-bound Fur has never been identified in *E. coli* or any other bacteria because of its weak binding activity and a very low intracellular free iron concentration (35). In searching for the putative iron-bound Fur in bacteria (22), we unexpectedly found that *E. coli* Fur binds a [2Fe-2S] cluster, but not a mononuclear iron, in the *E. coli* mutant cells in which intracellular free iron content is elevated due to deletion of the iron–sulfur cluster assembly proteins IscA and SufA (23). This notion has now been substantiated by the observations that *E. coli* Fur also binds a [2Fe-2S] cluster in wildtype *E. coli* cells grown in M9 medium supplemented with increasing concentrations of iron (Figs. 1 and 4). Furthermore, we find that binding of the [2Fe-2S] cluster in *E. coli* Fur turns on its Fur-box binding activity and that removal of the cluster eliminates its Fur-box binding activity (Fig. 3). It should be pointed out that the maximum occupancy of the [2Fe-2S] cluster in Fur is about 36% even in *E. coli* cells grown in M9 medium supplemented with excess iron (Fig. 4). Perhaps, intracellular free iron content is tightly regulated by an active Fur in such that only portion of Fur binds a [2Fe-2S] cluster and is active as a repressor in cells. In this context, we propose that *E. coli* Fur regulates intracellular iron homeostasis by reversibly binding a [2Fe-2S] cluster, but not a monocular iron, in response to elevation of intracellular free iron content in bacteria.

Site-directed mutagenesis studies have shown that *E. coli* Fur binds the [2Fe-2S] cluster at site 3, as mutation of Cys-93 or Cys-96 to Ala in site 3 results in Fur mutants that do not bind the [2Fe-2S] cluster (22). It turns out that both Fur mutants C93A and C96A have a diminished Fur-box binding activity *in vitro* and are inactive to complement the function of Fur *in vivo* (Fig. 5). The notion is consistent with the previous report showing that the *E. coli* Fur mutants with mutation of Cys-93 or Cys-96 to Ser (C93S or C96S) have a decreased Fur-box binding activity *in vitro* and are inactive *in vivo* (36). Taken together, these results strongly suggest that Cys-93 and Cys-96 are required for binding the [2Fe-2S] cluster in *E. coli* Fur and that binding of a [2Fe-2S] cluster is essential for the regulatory function of *E. coli* Fur (22). These cysteine residues are highly conserved among Fur proteins (2, 8). For example, the Fur homologs from *Haemophilus influenzae*, *Vibrio cholera* (13), and *H. pylori* (14) all have the conserved Cys-93 and Cys-96 and are able to bind a [2Fe-2S] cluster in the *E. coli* mutant cells with an elevated intracellular free iron content (26). Interestingly, the *Pseudomonas aeruginosa* Fur only has one cysteine residue, and the *Pseudomonas putida* Fur has no cysteine residues (37). Whether these Fur homologs can bind a [2Fe-2S] cluster or a mononuclear iron in response to elevation of intracellular free iron content remains to be investigated.

**Figure 6:**
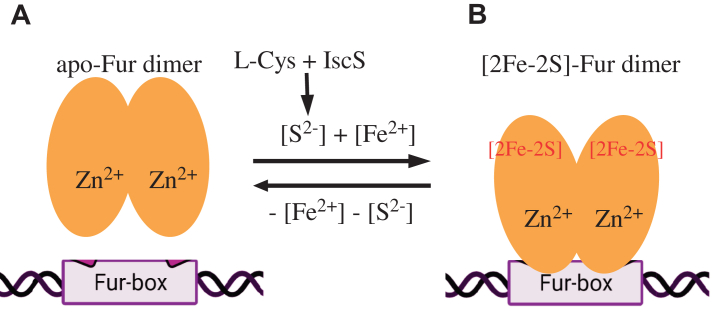
**A proposed model for Fur regulation in response to elevation of intracellular free iron content in *E. coli* cells.***A*, when intracellular free iron content is depleted, Fur does not bind any [2Fe-2S] clusters (Apo-Fur) and is inactive to bind the Fur-box. *B*, when intracellular free iron content is elevated, Fur assembles a [2Fe-2S] cluster from intracellular free Fe(II) and sulfide that is provided by L-cysteine and cysteine desulfurase (IscS). Fur becomes active to bind the Fur-box upon binding of a [2Fe-2S] cluster. A structural Zn(II) binding site is shown in both Apo-Fur and the [2Fe-2S] cluster-bound Fur. Fur, ferric uptake regulator.

Iron–sulfur clusters in proteins are assembled by a group of dedicated iron–sulfur cluster assembly proteins in bacteria (38, 39). Sulfide in iron–sulfur clusters is delivered by cysteine desulfurase and L-cysteine (40), while iron in the cluster is provided by intracellular iron content. Because the L-cysteine concentration in *E. coli* cells is about 200 μM under normal growth conditions (41), the intracellular free iron content is most likely the limiting factor for the [2Fe-2S] cluster assembly in Fur. It is envisioned that when intracellular free iron content is elevated, a [2Fe-2S] cluster is quickly assembled in Fur, and Fur becomes an active repressor to regulate intracellular iron homeostasis (Fig. 6). Unlike the binding of iron to Fur, which has a fairly weak binding affinity (18, 19, 20, 21), the enzymatic assembly of a [2Fe-2S] cluster in Fur will be more sensitive to increases in intracellular free iron content. On the other hand, while the oxidized [2Fe-2S] cluster in Fur is relatively stable, the reduced [2Fe-2S] cluster in Fur is unstable and quickly releases ferrous iron and sulfide, an unusual feature of the [2Fe-2S] cluster in Fur (26) (Fig. 3*A*). Thus, Fur may release iron and sulfide from the reduced [2Fe-2S] cluster in response to depletion of intracellular free iron (26) to become inactive apo-Fur (Fig. 6). However, the mechanism by which cluster reduction and dissociation is coupled to intracellular free iron content is not known and will be important for better understanding how Fur acts as an intracellular free iron sensor. While our data suggest that cluster reduction may be one potential mechanism that can release the cluster in response to low intracellular free iron content, there may be other as yet to be determined mechanisms. It should be pointed out that a number of gene transcription factors that bind an iron–sulfur cluster have been identified in *E. coli* and other bacteria (42, 43). For example, Fnr binds a [4Fe-4S] cluster to regulate anaerobiosis (44); SoxR binds a [2Fe-2S] cluster to control the genetic response to superoxide stress or nitric oxide stress (45, 46); and IscR becomes an active repressor upon binding of a [2Fe-2S] cluster to regulate iron–sulfur cluster biogenesis (47). Here, we propose that *E. coli* Fur binds a [2Fe-2S] cluster to regulate intracellular iron homeostasis in response to elevation of intracellular free iron content and that Fur represents a new member of the iron–sulfur cluster-containing transcription factor family in bacteria.

## Experimental procedures

### Protein purification

Plasmid pBAD expressing *E. coli* Fur or Fur mutants (Fur-C93A and Fur-C96A) was introduced into wildtype *E. coli* (MC4100 or GC4468) cells as described previously (22). Overnight *E. coli* cultures were inoculated 1:100 dilution in freshly prepared M9 medium supplemented with 20 amino acids (100 μg/ml), thiamine (0.1 μg/ml), glycerol (0.4%), Fe(NH_4_)_2_(SO_4_)_2_ (0–10.0 μM), and ampicillin (100 μg/ml). When the cells were grown to absorbance at 600 nm of 0.6 at 37 ^°^C under aerobic growth conditions, protein expression was induced by adding L-arabinose (0.04%). The cells were grown for additional 3 h before *E. coli* Fur was purified as described previously (22). The purity of purified Fur proteins was more than 90% as judged by electrophoresis analysis on a 15% polyacrylamide gel containing SDS followed by staining with Coomassie Blue. The concentration of purified *E. coli* Fur was measured at 280 nm after iron–sulfur clusters in the protein were removed by adding HCl (20 mM). The extinction coefficient of 5.6 mM^−1^ cm^−1^ at 280 nm was used for calculating the concentrations of purified *E. coli* Fur and the Fur mutant proteins C93A and C96A.

### Iron, sulfide, and zinc content determination

The amounts of iron and sulfide in Fur protein samples were analyzed according to the Fischer’s method (48) and the Siegel’s method (49), respectively, as described previously (22). Total Zn(II) content in Fur protein samples was determined using the Zn(II) indicator 4-(2-pyridylazo) resorcinol (PAR) (50). Purified *E. coli* Fur (50 μM) in buffer containing NaCl (500 mM) and Tris (20 mM, pH 8.0) was incubated with PAR (100 μM) at 80 ^°^C for 2 h, followed by centrifugation at 13,000 rpm for 10 min. The supernatant was subjected to UV-Vis absorption measurements. The extinction coefficient of 66 mM^−1^ cm^−1^ at 500 nm of the Zn–PAR complex (50) was used to calculate the Zn(II) content in Fur samples after subtracting the absorption amplitude of the Fe–PAR complex as described in (51).

### Removal of the mononuclear iron or the [2Fe-2S] cluster from *E. coli* Fur

To remove possible mononuclear iron from purified Red-Fur, the protein (50 μM) was incubated with EDTA (0.1 mM) at room temperature for 30 min before the dialysis against 2.0 L buffer containing NaCl (500 mM) and Tris (20 mM, pH 8.0) at 4 ^°^C for 4 h. To remove the [2Fe-2S] cluster from purified Red-Fur, the protein (50 μM) was incubated with freshly prepared sodium dithionite (4 mM) in 20 mM Tris (pH 8.0) at room temperature for 20 min before the dialysis against 2.0 L buffer containing NaCl (500 mM) and Tris (20 mM, pH 8.0) at 4 ^°^C for 4 h. The Slide-A-Lyzer MINI Dialysis Units (3.5K MWCO) (Thermo Scientific co) were used for the dialysis.

### Electrophoretic mobility shift assay

Two primers, Fur-A (5′-TTTAGGCGTGGCAATTCTATAATGA-3′ labeled with biotin at 5′-end) and Fur-B (5′-TATCAGTCATGCGGAATCTGTCCTG-3′) (Integrated DNA Technologies co), were used for the PCR amplification of the *E. coli fur* promoter region: (5′-TTTAGGCGTGGCAATTC**TATAATGATACGCATTATC**TCAAGAGCAAATTCTGTCACTTCTTCTAATGAAGTGAACCGCTTAGTAACAGGACAGATTCCGCATGACTGATA-3′) (110 bp). The highlighted sequence represents the consensus Fur-box (4). The biotin-labeled *fur* promoter fragment (0.7 nM) was incubated with increasing concentrations of Fur (0–2.0 μM) in 18 μl solutions containing Tris (22 mM, pH 8.0), glycerol (7%), MgCl_2_ (4.1 mM), KCl (44 mM), and NaCl (55 mM) at room temperature for 10 min and subjected to nondenaturing polyacrylamide gel (4%) electrophoresis. The biotin-labeled DNA fragments on the polyacrylamide gel were transferred to a nylon membrane (0.45 μm) (Thermo Fisher Scientific co), cross-linked under UV light at 120 mJ/cm^2^ for 1 min, and visualized using the Lightshift Chemiluminescence kit (Thermo Fisher Scientific co). The intensities of the Fur/DNA complex bands on the gel images were quantified using ImageJ (NIH).

### The *hin*fI site protection assay

The Fur-box binding activity of *E. coli* Fur was also analyzed using the *hin*fI site protection assay (18). Briefly, the Fur-box in the *E. coli iucABCD* promoter (5′-GA**GAATC**ATTAGCATTCGC-3′) which contains the restriction *hin*fI site (5′-GAATC-3′) was synthesized (GenScript co) and inserted into plasmid pUC19 *via Bam*HI and *Hin*dIII sites to create pUC19-*iuc*. Binding of Fur to the Fur-box protects the *hin*fI site from being cleaved by *Hin*fI (18). For the *hin*fI site protection assays, pUC19-*iuc* (3.2 nM) was preincubated with Fur proteins (0–2.0 μM) in 10 μl reaction solutions containing MgCl_2_ (2 mM), NaCl (150 mM), bovine serum albumin (0.1 mg/ml), and Tris (20 mM, pH 8.0) for 10 min at room temperature. Restriction enzyme *Hin*fI (1.0 unit) (New England Biolab co) was then added to the reaction solutions. After incubation at 37 ^°^C for 10 min, the reaction was stopped by adding 2 μl 6× loading buffer (New England Biolab co). The digested DNA products were separated by 1.5% agarose electrophoresis gel containing ethidium bromide (0.1 μg/ml) in 0.5X TAE (Tris-acetate-EDTA) buffer, run at 120 V for 35 min. The gel images were taken using the Kodak Gel Logic 200 Imaging System. The intensities of the DNA bands on the agarose gel images were quantified using ImageJ (NIH).

### Complementary assay of the *E. coli* Fur mutants

The gene encoding Fur was deleted in *E. coli* MC4100 strain using the one-step gene inactivation approach (31). Plasmid pBAD expressing either wildtype *E. coli* Fur or Fur mutant C93A or C96A was introduced into the *E. coli fur* mutant cells. Overnight culture of the *E. coli fur* mutant cells with pBAD expressing either wildtype Fur or the Fur mutant C93A or C96A was diluted 1:100 in freshly prepared M9 medium with either glycerol (0.4%) or succinate (0.4%). Cells were grown in the M9 media at 37 ^°^C under aerobic growth conditions for 20 h, and cell growth was measured from absorbance at 600 nm.

### Statistical analysis

All data are expressed as means ± standard deviations from at least three independent experiments.

## Data availability

All data generated and analyzed in the present study are included in the manuscript. Raw data are available on request.

## Supporting information

This article contains supporting information.

## Conflict of interest

The authors declare that they have no conflicts of interest with the contents of this article.
