# Supplementary material for: Ferric uptake regulator (Fur) binds a [2Fe-2S] cluster to regulate intracellular iron homeostasis in Escherichia coli
Source: J Biol Chem. 2023 Apr 24;299(6):104748. doi: 10.1016/j.jbc.2023.104748 (PMC10318462; doi:10.1016/j.jbc.2023.104748)

Supporting Information

Ferric uptake regulator (Fur) binds a [2Fe-2S] cluster to regulate intracellular iron homeostasis in *Escherichia coli*

Chelsey R. Fontenot, and Huangen Ding

Supplemental Figure 1. Restriction map of pUC19-*iuc*

Supplemental Figure 2, Fur binds a [2Fe-2S] cluster in E. coli GC4468 cells grown in M9 medium supplemented with increasing concentrations of iron

Supplemental Figure 1. Restriction map of pUC19-*iuc*

(A) Restriction map of pUC19-*iuc*. The underlined *hin*fI site is within the Fur-box.
(B) pUC19-iuc (3.4 nM) was incubated with restriction enzyme *Hinf*I (1.0 unit) in 10 µL reaction solution containing NaCl (150 mM), $MgCl_2$ (2 mM), BSA (0.1 mg/ml), and Tris (20 mM, pH 8.0) at 37°C for 10 min. The products were separated by 1.5% agarose electrophoresis gel. Lane 1, 1kb ladder. Lane 2, pUC19-*iuc* with no protein added. Lane 3, pUC19-*iuc* was pre-incubated with ferredoxin (2.0 µM). Lane 4, pUC19-*iuc* was pre-incubated with Red-Fur (2.0 µM). Lane 5, pUC19-*iuc* was first digested with *Hin*fI, followed by addition of Red-Fur (2.0 µM).

A

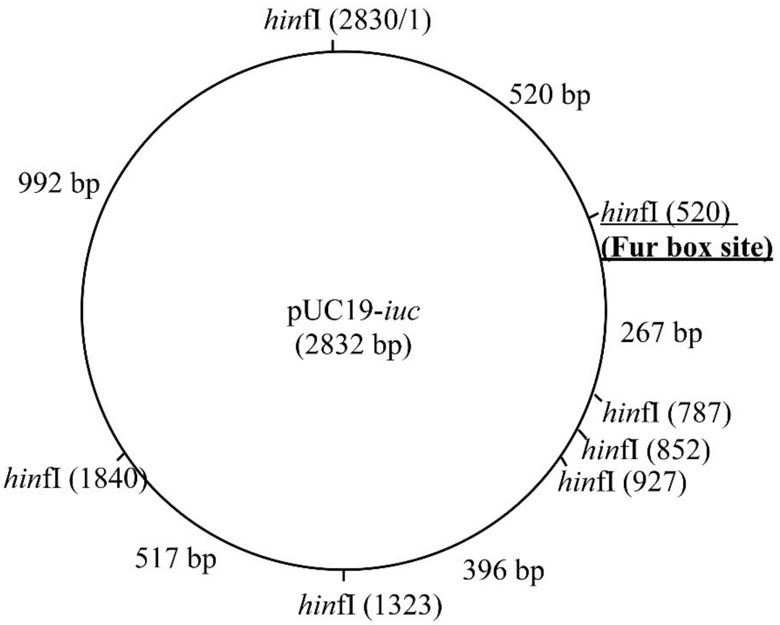

B

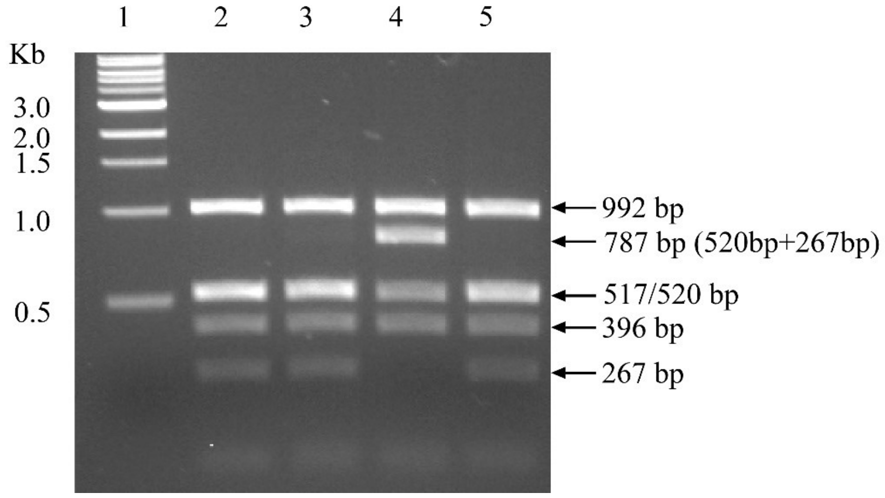

Supplemental Figure 2. Fur binds a [2Fe-2S] cluster in *E. coli* GC4468 cells grown in M9 medium supplemented with increasing concentrations of iron

(A) VU-Visible absorption spectra of purified Fur. Spectra 1-5, Fur purified from *E. coli* GC4468 cells grown in M9 medium supplemented with 0.0 μM, 0.5 μM, 1.0 μM, 2.0 μM or 10.0 μM $Fe(NH_4)_2(SO_4)_2$, respectively Purified Fur proteins (50 μM) were in buffer containing NaCl (500 mM) and Tris (20 mM, pH 8.0). **(B)** *E. coli* Fur binds the [2Fe-2S] cluster in wild type *E. coli* GC4468 cells in response to increasing concentrations of iron in M9 medium. The [2Fe-2S] cluster occupancy of Fur was calculated from the amplitude of the absorption peak at 410 nm (panel A) and plotted as a function of the iron concentrations in M9 medium.

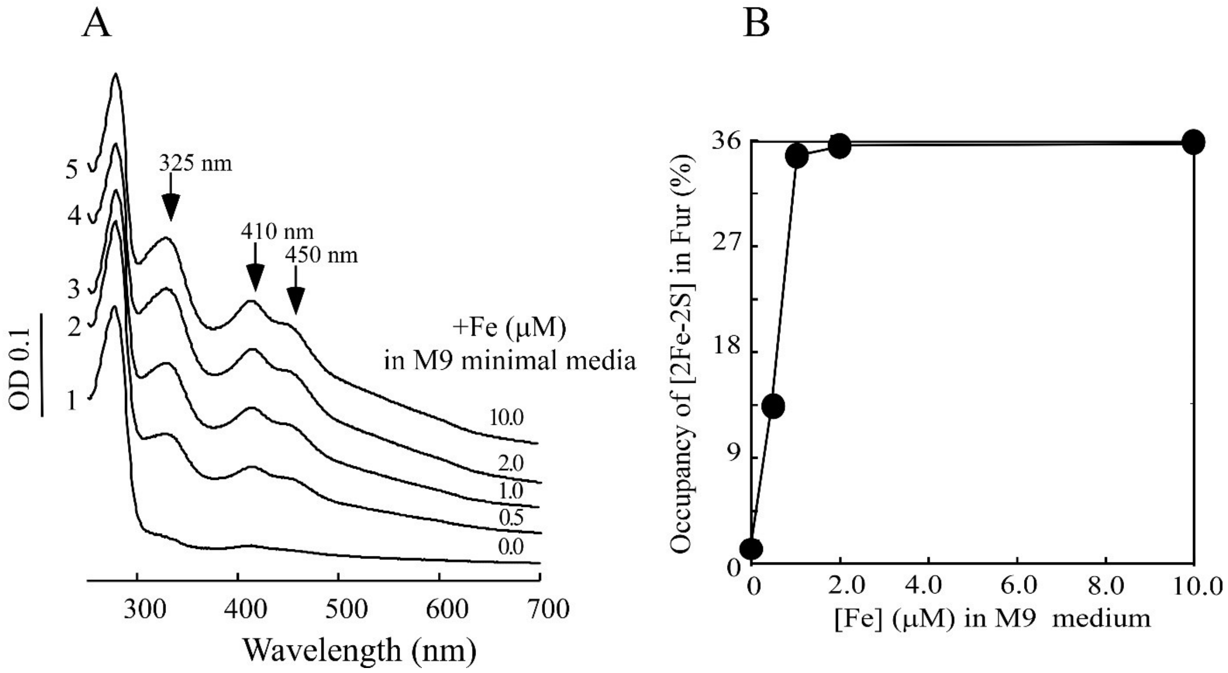

Supplement: Supporting Figures S1 and S2 [file mmc1.pdf]
